# Ictal asystole secondary to suspected herpes simplex encephalitis: a case report

**DOI:** 10.1186/1757-1626-2-9378

**Published:** 2009-12-22

**Authors:** Robert Gooch

**Affiliations:** 1 Schulich School of Medicine and Dentistry, The University of Western Ontario, 61 Gunn Street, London, ON N6G 1C6, Canada

## Abstract

Herpes simplex virus is a leading cause of sporadic encephalitis. While seizures are a common feature of Herpes simplex virus encephalitis, and periods of asystole have been reported in Herpes simplex virus patients, there have been no prior reports of ictal asystole secondary to such an infection.

This case report describes a 33 year old, previously healthy, gentleman of Malaysian descent, with new onset seizures resulting in a twenty-one second period of ictal asystole. In hospital the patient developed focal neurological symptoms. A diagnosis of Herpes simplex virus encephalitis was made, although this diagnosis was not confirmed by lumbar puncture, magnetic resonance imaging or biopsy.

Literature is reviewed regarding ictal asystole as well as clinical features and cardiac complications of Herpes simplex virus encephalitis. Given the link between ictal asystole and Herpes simplex virus encephalitis, cardiac monitoring would be recommended for Herpes simplex virus encephalitis patients having seizures. The use of anticonvulsants with cardiac side effects should be carefully considered.

## Introduction

Herpes simplex virus (HSV), usually type 1, is a leading cause of sporadic encephalitis, with an annual incidence of between 1 in 250 000 and 1 in 1 000 000 [1,2]. Common presenting symptoms and signs include headache, altered level of consciousness, focal neurologic symptoms, fever, aphasia, and seizures. The seizures associated with HSV infection are most often focal.

Cerebrospinal fluid (CSF) analysis tends to show pleocytosis, as well as increased protein and erythrocytes. Magnetic resonance imaging (MRI) is recommended if there is suspicion of HSV infection [1]. T2 weighted scans often show temporal lobe hyperintensity. HSV patients occasionally have normal scans. Auyeung and Dunn report a case of confirmed HSV encephalitis with normal MRI and no CSF leukocytosis [3]. Computed tomography (CT) is useful to rule out other causes for neurologic symptoms, such as mass lesions, and is recommended if a patient is unable to undergo MRI. Electroencephalogram (EEG) tends to show nonspecific findings, including periodic lateralising epileptiform discharges. Single photon emission computed tomography (SPECT) scans can be used as an adjunct; in HSV patients, the common finding is unilateral temporal lobe hypoperfusion [1].

The gold standard for HSV encephalitis diagnosis is polymerase chain reaction (PCR) detection of HSV DNA in the CSF. Sensitivity is 98% and specificity is 94% [2]. Brain biopsy is another option for diagnosis. However, virus distribution may not be uniform, leading to false negative results, and the procedure is invasive. It has a role when CSF is not available.

In addition to neurologic complications, HSV encephalitis has also been associated with cardiac arrythmias. Smith et al. report a case of a patient with HSV encephalitis confirmed by PCR who had short episodes of unconsciousness [4]. Electrocardiogram (ECG) monitoring showed that these were associated with bradycardia and periods of asystole lasting up to 7 seconds. Alsolaiman et al. report the case of a woman who presented with syncope and, while asleep in hospital, had asymptomatic asystolic periods of 4 to 8 seconds [5]. Findings of CSF analysis and MRI were consistent with HSV encephalitis. Proposed causes for such asystole include herpes simplex myositis and central nervous system (CNS) causes. There is no evidence that HSV has ever been found in the heart [5]. A central cause for the asystole in these cases, which may involve parasympathetic stimulation or sympathetic inhibition, would be in accordance with proposed mechanisms for ictal asystole (IA).

Asystole is a rare complication of focal seizures, but it is concerning, given its hypothesized link to sudden unexplained death in epilepsy (SUDEP). A study in a large surgical epilepsy centre found IA in 5 out of 1244 patients (0.4%); asystolic periods were as long as 60 seconds [6]. As patients with IA do not have asystole with every seizure, it is quite possible that this study did not record asystole in some patients with IA; the prevalence may, indeed, be significantly higher. In a group of 19 patients with intractable focal seizures, 4 had asystolic periods [7,8].

IA can present as a sudden loss of tone and a fall during a partial seizure. It is important, but may be clinically difficult, to differentiate IA from convulsive syncope or temporal lobe syncope. In convulsive syncope, the loss of consciousness and the fall tend to occur simultaneously, while in temporal lobe syncope a loss of tone is seen at the beginning of seizure activity. Atonia in IA patients occurs later in the seizure, and usually after an asystolic period of about 8 seconds [7]. The best method to differentiate between these entities seems to be simultaneous video/EEG/ECG monitoring [9].

The etiology of IA may be a result of seizure spread to autonomic centres resulting in sympathetic inhibition or parasympathetic stimulation. Attempts have been made to localize these centres. Experimenting on rats, Oppenheimer et al. found that stimulation of the rostral posterior insular cortex resulted in tachycardia while more caudal stimulation caused bradycardia, heart block, ventricular ectopics, and death [10,11]. In experiments on presurgical lobectomy patients, stimulation of the left insular cortex tended to cause bradycardia and a depressor response [10,12]. Patients with temporal lobe and insular infarction may have decreased heart rate variability [13-15].

Schuele et al., in a study involving combined EEG/ECG monitoring, found that there did not seem to be a distinct lateralization [7]. Indeed, at the onset of asystole in the patients with temporal lobe epilepsy, half the patients had bilateral temporal involvement, a quarter of the patients had left temporal involvement and the rest had right temporal involvement. This study did, however, demonstrate that IA tends to result from temporal lobe seizures. 8 out of the 10 patients with IA in the study had temporal lobe epilepsy. In the two patients without temporal lobe epilepsy, the asystolic periods may have been secondary to respiratory arrest. IA was not seen in patients with generalized epilepsy [7]. Britton et al. also found that temporal lobe seizures tended to cause bradyarrhythmias; 12 of 13 patients with ictal bradyarrhythmia had temporal lobe seizures. A review of the literature showed that of 65 patients with ictal bradyarrhythmia, 36 had temporal seizures and 15 had frontotemporal seizures [13].

## Case presentation

A 33-year-old, male, right handed, realtor, of Malaysian descent, presented to hospital following a nocturnal seizure. His wife reported being awoken by a cry and found her husband convulsing. The patient's arms were flexed and his eyes open and rolled back. This lasted for approximately 1 minute and was followed by restless thrashing movements and awkward attempts to get up. There was some foaming at the mouth.

On admission there was some residual disorientation but no lateralizing symptoms or signs. The patient had been previously well, although one week prior he had had flu-like symptoms, including loss of appetite and general myalgia, as well as a swelling on the right side of his neck which had resolved on its own.

There was no history of previous seizures, meningitis, encephalitis, blackouts, loss of consciousness, head injuries, or febrile seizures. The patient reported no use of recreational drugs. Neither the patient nor his family members had a history of tuberculosis. The patient's most recent travel was an Alaskan cruise. There were mosquitoes but the patient had not been bitten. He had not been bitten by any ticks or animals. There were no exposures to sick people. The patient denied any history of sexually transmitted infections or other relevant past illnesses.

In hospital, the patient was started on phenytoin but continued to have further nocturnal seizures with post ictal periods of approximately 30 minutes. During the third seizure there was a twenty-one second period of asystole and a permanent pacemaker was placed the following day.

Prior to the placement of the pacemaker, the patient was afebrile and had no headache, vision changes, or difficulty speaking or swallowing. There was no abnormality in bowel or bladder function. Gait was normal, and the patient's neurological exam was unremarkable except for gaze evoked nystagmus, thought to be a side effect of the phenytoin. Following the procedure, the patient reported feeling better and his wife reported that conversation with him was more normal, although he had forgotten details of events up to a week prior.

The same morning, however, the patient had a fourth seizure from sleep. The patient's phenytoin was optimized. Lumbar puncture (LP) showed WBC 7 cells/μL, RBC 1 cell/μL, protein 428 mg/L, and glucose 4.1 mmol/L. CT head with contrast was normal. The placement of the pacemaker precluded MRI.

Levetriacetam was added, but the patient continued to have recurrent seizures. He developed additional symptoms. There was a right sided headache, right ear pain and tenderness, and an unusual sound in the right ear, which evolved into a hallucination of music. The patient also developed complex partial seizures; these involved speech arrest, leftward head and eye turning, as well as left arm posturing. The focal neurologic symptoms and focal seizures suggested a diagnosis HSV encephalitis, despite the normal LP results. A course of acyclovir was started and further investigations were carried out.

Results of a second LP were also normal. PCR for Herpes simplex was negative. Cytology was negative, and repeat CT head with contrast was normal. SPECT showed a slight decrease in perfusion of the left side compared to the right. Repeat EEG showed nonspecific abnormality of the right temporal lobe. A biopsy was not done as there was a lack of a definable area to target.

Over the first week of acyclovir therapy the patient showed significant improvement, and the headache and seizures resolved. The hallucination of music persisted for several days. The patient was discharged to complete a 3 week course of acyclovir. Tapering of the anticonvulsants resulted in breakthrough seizures, so the patient remains on levetriacetam and phenytoin.

## Discussion

In the literature there are no reported cases of ictal asystole secondary to HSV encephalitis. The patient in the case presented here had a negative PCR and CSF analyses, and an MRI was unavailable due to the implantation of a pacemaker. However, the seizures, focal neurologic symptoms, evolution of focal seizures, and rapid improvement with initiation of acyclovir therapy strongly suggest a diagnosis of herpes simplex encephalitis.

HSV tends to affect the temporal lobes, which is also the localization of the majority of seizures associated with IA. Given that HSV has been associated with periods of asystole in patients without seizures, IA associated with HSV infection should not be entirely unexpected. The mechanism of the asystole in this case may indeed be a result of involvement of temporal autonomic centres. The trigeminocardiac reflex is also a consideration, as it describes a pathway wherein central stimuli can effect bradyarrhythmia and asystole [16,17]. Regardless of mechanism, cardiac monitoring may be warranted in HSV encephalitis patients, especially those having seizures. The use of anticonvulsants with cardiac side effects in such patients should be considered carefully.

## Competing interests

The author declares that he has no competing interests.

## Consent

Written informed consent was obtained from the patient for publication of this case report and accompanying images. A copy of the written consent is available for review by the Editor-in-Chief of this journal.
